# Bayesian neural networks with variable selection for prediction of genotypic values

**DOI:** 10.1186/s12711-020-00544-8

**Published:** 2020-05-15

**Authors:** Giel H. H. van Bergen, Pascal Duenk, Cornelis A. Albers, Piter Bijma, Mario P. L. Calus, Yvonne C. J. Wientjes, Hilbert J. Kappen

**Affiliations:** 1 SNN Machine Learning Group, Biophysics Department, Donders Institute for Brain Cognition and Behavior, Radboud University, 6525 AJ Nijmegen, The Netherlands; 2 Animal Breeding and Genomics, Wageningen University and Research, 6700 AH Wageningen, The Netherlands; 3 Department of Molecular Developmental Biology, Radboud Institute for Molecular Life Sciences, Radboud University, 6500 HB Nijmegen, The Netherlands; 4 Department of Human Genetics, Donders Institute for Brain, Cognition and Behaviour, Radboud University Medical Center, 6500 HB Nijmegen, The Netherlands; 5 Present Address: Euretos B.V., Yalelaan 1, 3584 CL Utrecht, The Netherlands

## Abstract

**Background:**

Estimating the genetic component of a complex phenotype is a complicated problem, mainly because there are many allele effects to estimate from a limited number of phenotypes. In spite of this difficulty, linear methods with variable selection have been able to give good predictions of additive effects of individuals. However, prediction of non-additive genetic effects is challenging with the usual prediction methods. In machine learning, non-additive relations between inputs can be modeled with neural networks. We developed a novel method (NetSparse) that uses Bayesian neural networks with variable selection for the prediction of genotypic values of individuals, including non-additive genetic effects.

**Results:**

We simulated several populations with different phenotypic models and compared NetSparse to genomic best linear unbiased prediction (GBLUP), BayesB, their dominance variants, and an additive by additive method. We found that when the number of QTL was relatively small (10 or 100), NetSparse had 2 to 28 percentage points higher accuracy than the reference methods. For scenarios that included dominance or epistatic effects, NetSparse had 0.0 to 3.9 percentage points higher accuracy for predicting phenotypes than the reference methods, except in scenarios with extreme overdominance, for which reference methods that explicitly model dominance had 6 percentage points higher accuracy than NetSparse.

**Conclusions:**

Bayesian neural networks with variable selection are promising for prediction of the genetic component of complex traits in animal breeding, and their performance is robust across different genetic models. However, their large computational costs can hinder their use in practice.

## Background

The biochemical mechanisms that underlie phenotypes work through non-linear interactions between molecules and proteins. Nevertheless, in the practice of animal breeding, additive prediction methods, which assume that phenotypes depend on markers individually and without interactions between them, have been successful. The U-shaped allele frequency of causal loci explains why these microscopic interactions give rise to traits mainly due to additive genetic variance [1], and therefore the success of additive methods. However, traits still have an epistatic component and therefore methods that can fit more than the additive genetic component have the potential to better predict genotypic values and phenotypes of animals.

In reality, the causal variants of a trait are not necessarily among the markers that are used for genomic prediction. Therefore, not all markers may aid in the prediction of genetic values. Prediction methods may therefore be optimized if they allow to model a proportion $\pi$ of the total number of markers as irrelevant for phenotype prediction. Additive methods such as BayesB [2] and BayesC$\pi$ [3] allow for this variable selection of markers (sparsity). Depending on the genetic architecture of the trait, additive Bayesian variable selection methods may have a small advantage over genomic best linear unbiased prediction (GBLUP) [4–6].

Parametric methods assume a specific functional form. Members of this family of methods are the additive and dominance methods of quantitative genetics. In addition, parametric methods assume that genetic variance can be decomposed orthogonally into additive, dominance, additive $\times$ additive, dominance $\times$ additive etc. variance components. This orthogonal decomposition is valid only under restricted assumptions such as linkage equilibrium, random mating and no inbreeding [7]. Since these assumptions are invalid in practice, prediction methods that do not make them have the potential to obtain better predictive performance of traits than methods that do.

Non-parametric methods assume neither a particular form of the unknown relation from genetic material to genotypic values, nor the aforementioned partitioning of genetic variance. Because these models do not distinguish between the different variance components, they predict genotypic values instead of breeding values, where breeding values correspond only to the additive component of genotypic values. In genetics, reproducing kernel Hilbert space regression [8] and neural networks have been studied as non-parametric methods for the prediction of phenotypes. Neural networks, in particular, are powerful and interesting non-parametric methods because they can approximate any function, and current software packages make them easy to use.

Neural network models that have been investigated in animal breeding include Bayesian regularized artificial neural network (BRANN) [9, 10], scaled conjugate gradient artificial neural network (SCGANN) [10] and approximate Bayesian neural network [11] methods. BRANN is a neural network method that avoids overfitting by means of Bayesian regularization via Bayesian prior distributions, and achieved higher accuracy than additive methods for prediction of milk production in Jersey cows. For marbling score in Angus cattle, BRANN had a higher predictive accuracy than both additive methods and SCGANN, which is a neural network method without Bayesian regularization. These results imply that Bayesian regularization can have a benefit for prediction of traits. BRANN has been used for the ranking of markers based on their impact on the network. [12] While this approach can help to identify the most important markers, it does not promote sparsity during inference since the ranking is performed as a separate step after inference.

There are several neural network methods that try to achieve sparsity with different regularizations on the weights (parameters of the neural network). For example, $\ell _1$ (Lasso) regularization causes as many weights as possible to reach zero and has previously been studied in animal breeding [13], and group Lasso, which allows for pruning weights in groups instead of individually, has been studied for image classification [14]. Sparsity based on an $\ell _0$ regularization for individual weights has also been studied for image classification [15]. These approaches are based on Maximum a Posteriori inference, and typically focus on sparsity of all network nodes or weights, rather than sparsity of inputs (markers) specifically.

In summary, there are phenotype prediction methods that allow for variable selection of markers and there are non-parametric methods that allow for fitting non-additive effects of individuals. However, there are no methods that allow for both. In this study, we introduce a method called NetSparse to fill the previously unexplored combination of both Bayesian variable selection and Bayesian neural networks for prediction of total genotypic values.

In the section Methods, the framework for Bayesian phenotype prediction is set and we explain how both additive Bayesian methods and NetSparse fit within this framework. The section Simulations describes the simulation of data used to compare NetSparse with other methods. In the section Results, we compare NetSparse to the reference methods GBLUP, BayesB, GBLUP-AD, BayesB-AD and GBLUP-ADAA.

## Methods

First, we set up a general Bayesian framework for phenotype prediction methods, then we will describe how the different methods considered fit in this framework.

### Bayesian phenotype prediction

We assume that *N* individuals $(i=1,\ldots ,N)$ are phenotyped and genotyped, such that the observed phenotype $y_i$ is the sum of a genotypic value $g_i$ and a residual $e_i$. In addition, we assume that the genotypic value of individuals can be computed from their marker genotypes by a function $f(\cdot ; \mathbf {u})$, depending on unknown parameters $\mathbf {u}$ (different for each method), and that the residuals $e_i$ follow a normal distribution with mean 0 and variance $\sigma _e^2$, which we write as $e_i\sim \mathcal {N}\left( 0,\sigma _e^2\right)$. These assumptions lead to the following model:

1 $$y_i = f\left( \bf {x}_i;\bf {u}\right) + e_i, $$

where $\mathbf {x}_i=\left( x_i^1\,x_i^2\,\ldots \,x_i^P\right) ^{\intercal }$ is the vector with marker genotypes of individual *i*, the exact encoding of which depends on the method. We gather the vectors in the matrix $\mathbf {X}=\left( \mathbf {x}_1\,\mathbf {x}_2\,\cdots \,\mathbf {x}_N\right)$. Model (1) implies that the likelihood of the data is:

$$\begin{aligned} p\left( \mathbf {y}\mid \mathbf {X}, \mathbf {u}, \sigma _e^2\right)&= \prod _{i=1}^N\mathcal {N}\left( y_i\mid f\left( \mathbf {x}_i;\mathbf {u}\right) , \sigma _e^2\right) \\&=\left( 2\pi \sigma _e^2\right) ^{-N/2}\exp \left( -\frac{1}{2\sigma _e^2}\sum _{i=1}^{N}\left( y_i - f(\mathbf {x}_i;\mathbf {u})\right) ^2\right) . \end{aligned}$$

The likelihood is combined with a prior distribution $p\left( \mathbf {u}\mid \eta \right)$, depending on hyperparameters $\eta$, which indicates what a priori are considered to be plausible values of $\mathbf {u}$ that could have generated our data.

In our terminology, we use the term “parameter” for $\mathbf {u}$, these are variables which directly determine the model predictions. The term “hyperparameter” is used for $\eta$, these are the parameters which only indirectly influence the predictions of the model. The hyperparameters $\eta$ and $\sigma _e^2$ can crucially influence the performance of a model. There are roughly two approaches for these hyperparameters: they can be either estimated or integrated out. For the GBLUP models in particular, where the hyperparameters are the variance components,^1^ this estimation can be done for instance with restricted maximum likelihood (REML) [16]. Alternatively, if estimation of these hyperparameters is difficult, they can be given prior distributions and integrated out together with the other parameters via Markov chain Monte Carlo sampling (“MCMC sampling” section).

The joint distribution of $\mathbf {y}$ and $\mathbf {u}$ conditioned on $\mathbf {X}$ is the product of the likelihood and prior distribution:

$$\begin{aligned} p\left( \mathbf {y}, \mathbf {u}\mid \mathbf {X}\right) = p\left( \mathbf {y}\mid \mathbf {X}, \mathbf {u}\right) p\left( u\right) . \end{aligned}$$

The posterior distribution $p\left( \mathbf {u}\mid \mathbf {X},\mathbf {y}\right)$ via Bayes’ Theorem is then:

2 $$\begin{aligned} p\left( \mathbf {u}\mid \mathbf {X},\mathbf {y}\right) = \frac{p\left( \mathbf {y}\mid \mathbf {X}, \mathbf {u}\right) p\left( \mathbf {u}\right) }{\int p\left( \mathbf {y}\mid \mathbf {X}, \mathbf {u}'\right) p\left( \mathbf {u}'\right) \,\text {d}\mathbf {u}'}. \end{aligned}$$

This posterior distribution is the distribution over $\mathbf {u}$ obtained by combining the information in the prior distribution and the information in the observed data.

Model (1) can be used to make phenotype predictions $y_*$ for an individual with markers $\mathbf {x}_*$ by computing the posterior predictive distribution:

$$\begin{aligned} p\left( y_*\mid \mathbf {X},\mathbf {y},\mathbf {x}_*\right) = \int \mathcal {N}\left( y_*\mid f\left( \mathbf {x}_*;\mathbf {u}\right) , \sigma _e^2\right) p\left( \mathbf {u}\mid \mathbf {X}, \mathbf {y}\right) \,\text {d}\mathbf {u}. \end{aligned}$$

The expected value of $y_*$ with respect to the posterior predictive distribution is:

3 $$\begin{aligned} \mathbb {E}\left[ y_*=f\left( \mathbf {x}_*; \mathbf {u}\right) \right]&= \int y_*\, p\left( y_*\mid \mathbf {X},\mathbf {y},\mathbf {x}_*,\sigma ^2_e\right) \, \text {d}y_*\nonumber \\&= \int y_*\, \mathcal {N}\left( y_*\mid f\left( \mathbf {x}_*;\mathbf {u}\right) , \sigma _e^2\right) \nonumber \\&\quad p\left( \mathbf {u}\mid \mathbf {X}, \mathbf {y}\right) \,\text {d}\mathbf {u}\,\text {d}y_*\nonumber \\&= \int f\left( \mathbf {x}_*; \mathbf {u}\right) p\left( \mathbf {u}\mid \mathbf {X},\mathbf {y}\right) \,\text {d}\mathbf {u}. \end{aligned}$$

### Bayesian inference

Using the previous framework, we will briefly describe the five methods that we used for reference (GBLUP, BayesB, GBLUP-AD, BayesB-AD and GBLUP-ADAA), as well as our new method, NetSparse. Of these methods, GBLUP [17] was chosen because it is the most used in practice. We chose BayesB [2] because it is a common method that includes variable selection, similar to NetSparse.

#### GBLUP

The SNP-BLUP model is an additive model where each marker *p* is assigned an additive effect $a_p$ with shared prior variance $\sigma _a^2$. Specifically, in SNP-BLUP, *f* is chosen as a linear function $f_{\text {SNP-BLUP}}\left( \mathbf {x}; \mathbf {u}\right) = \mathbf {x}^{\intercal }\mathbf {a}$, with $\mathbf {u} =\mathbf {a}$. The prior distribution over $\mathbf {a}$ is $p\left( \bf {a}\mid \sigma _a^2\right) = {\mathcal {N}}\left( \bf {a}\mid \bf {0},\sigma _a^2\bf {1}\right)$. The hyperparameters $\sigma _e^2, \sigma _a^2$ are estimated with REML.

The posterior distribution over $\mathbf {a}$ is:

$$\begin{aligned} p\left( \mathbf {a}\mid \mathbf {X},\mathbf {y}\right) = \mathcal {N}\left( \mathbf {a}\mid \sigma _a^2\mathbf {\Sigma } \mathbf {X}\mathbf {y}, \mathbf {\Sigma }\right) , \end{aligned}$$

where $\mathbf {\Sigma }^{-1}=\sigma _e^2 \mathbf {1}+ \sigma _a^{2}\mathbf {X}^{\intercal }\mathbf {X}$. The matrix $\mathbf {X}^{\intercal } \mathbf {X}$ is proportional to the additive genomic relationship matrix $\mathbf {G}$. The posterior predictive distribution is also Gaussian:

4 $$\begin{aligned} p\left( y_*\mid \mathbf {X},\mathbf {y}, \mathbf {x}_*\right) = \mathcal {N}\left( y_*\mid \sigma _e^{-2}\mathbf {x}_*^{\intercal }\mathbf {\Sigma } \mathbf {X}\mathbf {y}, \mathbf {x}_*^{\intercal }\mathbf {\Sigma }\mathbf {x}_*\right) . \end{aligned}$$

This equivalent formulation of SNP-BLUP is called GBLUP and uses the additive relationship matrix $\mathbf {G}$ instead of allele effects, based on markers. For derivations of these formulas, see for instance [18]. We used the GBLUP implementation of MTG2 [19].

#### BayesB

The BayesB model, like SNP-BLUP, is an additive model where every marker is assigned an additive effect [2]. However, contrary to SNP-BLUP, BayesB also includes marker selection, which we will indicate by a (binary) marker selection vector $\mathbf {s}\in \{0,1\}^P$. If an entry in this marker selection vector has the value 1, the corresponding marker is selected for inclusion in the model. If the entry is equal to 0 the marker is not selected. If the posterior distribution is concentrated at $s^p=1$, then the *p*-th marker contributes significantly to phenotype prediction, while if most of the probability is concentrated at $s^p=0$ then the *p*-th marker does not contribute significantly to phenotype prediction. In addition, there is a hyperparameter $\pi$ that is equal to the proportion of non-contributing markers, i.e. $\pi =1-\sum _p s^p/P$. SNP-BLUP, in contrast, assumes that all additive effects come from the same normal distribution, making it similar, but not exactly equal, to BayesB with $\pi =0$. If a dataset contains relatively few quantitative trait loci (QTL), this mismatch should result in BayesB having a better performance than GBLUP.

Specifically, in BayesB, the function *f* is $f_{\text {BayesB}}\left( \mathbf {x}; \mathbf {u}\right) = \mathbf {a}^{\intercal } \left( \mathbf {x}\odot \mathbf {s}\right)$, with $\mathbf {u}=\left( \mathbf {a}, \mathbf {s}\right)$ and $\odot$ the element-wise (Hadamard) product $\left( \mathbf {x}\odot \mathbf {s}\right) ^p = x^ps^p$. For each marker *p*, marker effect $a_p$ has prior distribution $p\left( a_p\mid \sigma _{a_p}^2\right) =\mathcal {N}\left( a_p\mid 0,\sigma _{a_p}^2\right)$ and hyperprior distribution $p\left( \sigma _{a_p}^2\right) =\chi ^{-2}\left( \sigma _{a_p}^2\mid \text {df}=5, S=\frac{3}{5}\right)$, $p\left( s^p\mid \pi \right) = \pi ^{1-s^p}(1-\pi )^{s^p}$, and $p(\pi )\,=\,\text {Unif}(\pi \mid 0,1)$. The expression for the posterior distribution over $\mathbf {u}$ is:

5 $$\begin{aligned} p\left( \mathbf {a}, \mathbf {s}\mid \mathbf {X},y,\sigma _e^2\right) \propto \int&\frac{\exp \left( -\frac{\left\| \mathbf {y}- \mathbf {a}^{\intercal }\left( \mathbf {X}\odot \mathbf {s}\right) \right\| ^2}{2\sigma _e^2}\right) }{\left( \sigma _e^2\right) ^{N/2}}\nonumber \\&\prod _p\frac{\exp \left( -\frac{a_p^2}{2\sigma _{a_p}^2}\right) }{\sqrt{\sigma _{a_p}^2}}\chi ^{-2}\left( \sigma _{a_p}^2\mid 5,\frac{3}{5}\right) \nonumber \\&\pi ^{\sum _p 1-s^p}(1-\pi )^{\sum _p s^p}\, \text {d}\pi \, \text {d}\sigma _{a_p}^2 \end{aligned}$$

and the expected value of the posterior predictive distribution is^2^:

6 $$\begin{aligned} \mathbb {E}\left[ y_*\right]&= \sum _{\mathbf {s}}\int \left[ \mathbf {a}^{\intercal }\left( \mathbf {x}_*\odot \mathbf {s}\right) \right] p\left( \mathbf {a}, \mathbf {s}\mid \mathbf {X},\mathbf {y},\sigma _e^2\right) \,\text {d}\mathbf {a}\nonumber \\&= \left( \sum _{\mathbf {s}}\int \left( \mathbf {a}\odot \mathbf {s}\right) p\left( \mathbf {a}, \mathbf {s}\mid \mathbf {X},\mathbf {y},\sigma _e^2\right) \,\text {d}\mathbf {a}\right) ^{\intercal }\mathbf {x}_*. \end{aligned}$$

The last line comes from the identity $\mathbf {a}^{\intercal }\left( \mathbf {x}_*\odot \mathbf {s}\right) =\left( \mathbf {a}\odot \mathbf {s}\right) ^{\intercal }\mathbf {x}_*$ and means that prediction can be obtained by averaging allele effects and then making predictions using those, instead of averaging predictions directly. The expectation value cannot be computed analytically, but it can be approximated by sampling (“MCMC sampling” section).

### AD methods

The aforementioned additive methods can be adapted to fit additive and dominance effects. For the additive effects, markers were encoded as:

7 $$\begin{aligned} {\left\{ \begin{array}{ll} -\left( -p_{Aa}-2p_{aa}\right) \\ -\left( 1-p_{Aa}-2p_{aa}\right) \\ -\left( 2-p_{Aa}-2p_{aa}\right) \end{array}\right. }\text { for genotypes } {\left\{ \begin{array}{ll} AA\\ Aa\\ aa\end{array}\right. }, \end{aligned}$$

and for the dominance effects, markers were encoded as:

8 $$\begin{aligned} {\left\{ \begin{array}{ll} \displaystyle \frac{2p_{Aa}p_{aa}}{p_{AA}+ p_{aa}- \left( p_{AA}- p_{aa}\right) ^2}\\ \displaystyle \frac{4p_{AA}p_{aa}}{p_{AA}+ p_{aa}- \left( p_{AA}- p_{aa}\right) ^2}\\ \displaystyle \frac{2p_{AA}p_{Aa}}{p_{AA}+ p_{aa}- \left( p_{AA}- p_{aa}\right) ^2} \end{array}\right. }\text { for genotypes } {\left\{ \begin{array}{ll}AA\\ Aa\\ aa\end{array}\right. }, \end{aligned}$$

Note that for the GBLUP implementation and assuming Hardy-Weinberg equilibrium (HWE), the use of these encodings leads to additive and dominance relationship matrices as described in [20]. For the BayesB implementation, the two encodings are appended, such that every individual is represented by an array twice as long as for the additive models. Using GBLUP and BayesB with these longer arrays allows dominance to be fitted as well and we call the resulting methods GBLUP-AD and BayesB-AD [21, 22]. Because they explicitly model additive and dominance effects, these methods should work best on data where both additive variance and dominance variance are significant.

### GBLUP-ADAA

The AD construction for GBLUP can be extended further to fit additive by additive epistasis (section Simulations), in addition to additive and dominance effects, by adding a third covariance matrix, given by $\mathbf {G}\odot \mathbf {G}$. We call this method GBLUP-ADAA. As with GBLUP, the MTG2 software was also used for GBLUP-AD and GBLUP-ADAA.

### NetSparse

**Fig. 1:**
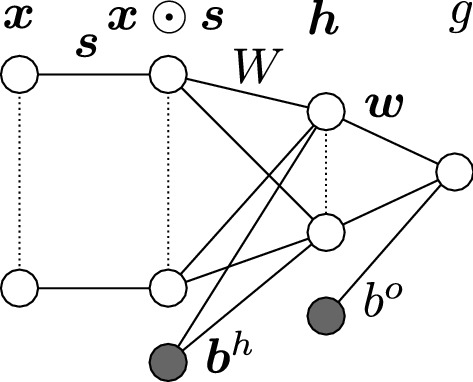
NetSparse Schematic neural network representation of NetSparse (9). The input $\mathbf {x}$ to the neural network is on the left, the output *g* is on the right. $\mathbf {s}$ is the variable selection vector, $\mathbf {W}$ and $\mathbf {w}$ are the weights, $\mathbf {b}^h$ and $b^o$ are the biases. At the third layer of nodes, $\tanh$ is applied to the sum of the incoming values

In our NetSparse model (Fig. 1), *f* is chosen as a neural network with one hidden layer^3^:

9 $$\begin{aligned} f_{\text {NetSparse}}\left( \mathbf {x}; \mathbf {u} \right) & = g \left( b^o + \mathbf {w}^{\intercal } \tanh \left( \mathbf {h}\left( \mathbf {x} \right) \right) \right) \text {, where} \end{aligned}$$

10 $$\begin{aligned} \mathbf {h}\left( \mathbf {x}\right)&= \mathbf {b}^h + \mathbf {W} \left( \mathbf {x}\odot \mathbf {s}\right) \end{aligned}$$

with $\mathbf {u}=\left( \mathbf {W},\mathbf {w}, \mathbf {b}^h, b^o, \mathbf {s}\right)$. $f_{\text {NetSparse}}$ is the output of the entire network, which depends on $\mathbf {h}(\mathbf {x})$, which is called the hidden layer. The vector $\mathbf {s}$ is a marker selection vector, like in BayesB. The parameters $\mathbf {W}\in {\mathbb {R}}^{H\times P}$ and $\mathbf {w}\in {\mathbb {R}}^H$ are called the weights, $\mathbf {b}^h\in {\mathbb {R}}^H$ and $b^o$ are called the biases. Parameter *H* is the number of hidden units and by increasing it, the neural network has more capacity to fit non-additive effects. In this study, as is typical for prediction of continuous outcomes, the output activation function *g* was chosen as the identity. For classification, a different transfer function, such as softmax, would be more appropriate, but such analyses fall outside the scope of this study. Given that the computational resources are sufficient, one would determine *H* via a cross-validation procedure. However, we did not have access to such resources, thus we used $H=20$, such that the model was able to fit complex non-linear interactions within reasonable computation time. A value of *H* larger than 20 led to an impractical increase in computation time.

A neural network can be interpreted as repeatedly taking linear combinations and elementwise application of an activation function ($\tanh$). Given $\mathbf {x}\odot \mathbf {s}$, the value of $h_i$ depends linearly on the *j*-th column of $\mathbf {W}$, and given each $\tanh \left( h_i\right)$, the output of the network depends linearly on $\mathbf {w}$. The non-linearity from $\tanh$ makes sure that the neural network can fit more than additive relations; if $\tanh$, as well as *g*, was replaced by the identity function the network would only be able to fit linear functions.

Some of the prior distributions are:^4^

$$\begin{aligned} W_{ij}\mid \sigma _h&\sim \mathcal {N}\left( 0,\sigma _h^2/P\right) ,\\ \mathbf {w}\mid \sigma _o&\sim \mathcal {N}\left( 0,\sigma _o^2/H\mathbf {1}\right) ,\\ s^p\mid \pi&\sim \text {Ber}(1-\pi ),\\ \pi&\sim \text {Unif}\left( 0,1\right) \text {, and}\\ \beta , \sigma _h, \sigma _o&\sim \left| \mathcal {N}\left( 0,2^2\right) \right| , \end{aligned}$$

where $\left| \mathcal {N}\left( 0,2^2\right) \right|$ denotes the half-normal distribution with scale parameter $2^2$, which is the same as a normal distribution with standard deviation 2 restricted to positive values only, and $\beta$ is the precision parameter in the (Gaussian) likelihood. The prior distribution over $\mathbf {s}$ is the same as that of BayesB. The posterior distribution over $\mathbf {u}$ in NetSparse (2) is:

11 $$\begin{aligned} p\left( \mathbf {u}\mid \mathbf {X},y\right) \propto \int&\frac{\exp \left( -\frac{\beta \left\| \mathbf {y}- \mathbf {w}^{\intercal }\tanh \left( \mathbf {W}\left( \mathbf {X}\odot \mathbf {s}\right) + \mathbf {b}^h\right) - b^o \right\| ^2}{2}-\frac{P\left\| \mathbf {W}\right\| ^2}{2\sigma _h^2}-\frac{H \left\| \mathbf {w}\right\| ^2}{2\sigma _o^2} - \frac{\sigma _h^2 + \sigma _o^2 + \beta ^2}{8} - \frac{\left\| \mathbf {b}^h - \varvec{\upmu }_{b_h}\right\| ^2}{2\sigma _{b_h}^2}\right) }{\beta ^{-N/2}\left( \sigma _h^2/P\right) ^{PH/2}\left( \sigma _o^2/H\right) ^{H/2}}\nonumber \\&\prod _p\pi ^{1-s^p}(1-\pi )^{s^p} \, \text {d}\pi \, \text {d}\sigma _o\, \text {d}\sigma _h\,\text {d}\beta . \end{aligned}$$

As with BayesB, this expression can not be computed analytically, but it can be approximated by sampling.

### MCMC sampling

The integral in (3) can be computed analytically for GBLUP, but not for BayesB (5) and NetSparse (11). To obtain an approximation to $\mathbb {E}\left[ y_*\right]$ for these models, we do MCMC sampling to obtain samples from the joint posterior distribution over $\left( \mathbf {u},\eta \right)$. Given such samples $\left( \left( \mathbf {u}_1,\eta _1\right) , \left( \mathbf {u}_2,\eta _2\right) , \ldots , \left( \mathbf {u}_T,\eta _T\right) \right)$, the expectation value of $y_*$ can be estimated as:

$$\begin{aligned} \mathbb {E}\left[ y_*\right] \approx \frac{1}{T}\sum _{t=1}^T f\left( \mathbf {x}_*; \mathbf {u}_t\right) . \end{aligned}$$

For BayesB, we implemented a Gibbs sampler in the BGLR R package [23]. Instead of averaging predictions, the sampler averages allele effects, but this is equivalent (see (6)).

For NetSparse, we used the PyMC3 package [24] to sample from the NetSparse posterior distribution, $p\left( \mathbf {u},\eta |\mathbf {X},\mathbf {y}\right)$, which is the integrand of (11). The conditional distributions cannot be sampled from, directly, so a Gibbs sampler cannot be used, therefore PyMC3 uses a composite sampler, which alternatively uses MCMC samplers for the discrete ($s^p$) and for the continuous variables (the rest). To sample $\mathbf {s}$, we used a Metropolis-Hastings algorithm, where we iterate over the individual components in a random order. For each $s^p$, we evaluate $P_1=p\left( s^p\mid \text {rest}\right)$ and $P_2=p\left( 1-s^{p}\mid \text {rest}\right)$, then we set $s^{p}\leftarrow 1-s^{p}$ with probability $\min (1,P_2/P_1)$.

For the continuous parameters, we have the conditional posterior distribution $p\left( \theta \mid \mathbf {X},\mathbf {y},\mathbf {s}\right)$, where we write $\theta$ for the combination of all continuous variables: $\mathbf {W},\mathbf {w}, \mathbf {b}^h, b^o,$ and $\eta$. This conditional posterior distribution is the integrand of (11). To sample these parameters, we used the Hamiltonian Monte Carlo sampler (HMC) [25, 26]. HMC uses the same Metropolis-Hastings procedure as for $\mathbf {s}$, but with a more complicated proposal. To generate a proposal, initialize $\mathbf {\theta }(0)\leftarrow \mathbf {\theta }$, and for each $\theta _i$ draw a new variable $r_i(0)$ from a normal distribution and compute the energy $E_0=H\left( \mathbf {\theta }(0), \mathbf {r}(0)\right) =\left\| \mathbf {r}(0)\right\| ^2/2-\log p\left( \mathbf {\theta }(0)\mid \text {rest}\right)$. Given this initial state, generate a proposal state from $(\mathbf {\theta }(0), \mathbf {r}(0))$ by numerically evolving it for a time *T* according to the Hamiltonian dynamics:

$$\begin{aligned} \frac{\text {d}\theta _i}{\text {d}t}&= r_i\\ \frac{\text {d}r_i}{\text {d}t}&= \frac{\partial \log p\left( \mathbf {\theta }\mid \text {rest}\right) }{\partial \theta _i}. \end{aligned}$$

This new state $\left( \mathbf {\theta }(T),\mathbf {r}(T)\right)$ will have energy $E_T=H\left( \mathbf {\theta }(T),\mathbf {r}(T)\right)$. This proposal is evaluated with a Metropolis-Hastings acceptance criterion: set $\mathbf {\theta }\leftarrow \mathbf {\theta }(T)$ with probability $\min \left( 1,\exp \left( E_0-E_T\right) \right)$, otherwise $\mathbf {\theta }\leftarrow \mathbf {\theta }(0)$. The $r_i$ are discarded. We note that only the gradient of the posterior distribution is required, but not the matrix of second derivatives.

We used the NUTS variant of HMC [27]. For high-dimensional models with continuous variables, using the gradient of the posterior distribution allows HMC to explore the parameter space faster than either Metropolis-Hastings or Gibbs [28] samplers [29], and therefore requires fewer sampler steps.

Besides computing the posterior distribution, simulation of the Hamiltonian dynamics also requires the gradient of the posterior distribution. PyMC3 calculates this gradient by the automatic differentiation capabilities of Theano [30].

We drew four independent chains of 1000 samples each, where for each chain the first 500 samples were used to tune the sampler and discarded, the last 500 samples of each chain were used for predictions. We also ran a few longer chains, but this did not change the results.

## Simulations

To compare the performance of these methods, we evaluated them on populations in which the traits have different phenotypic models (additive, dominance and epistatic).

### Population structure

Our aim was to simulate a population with a family structure and linkage disequilibrium pattern that roughly resemble those of livestock populations, using QMSim [31]. The historical population was simulated by mating 250 males with 250 females for 1900 generations to reach mutation-drift equilibrium. To mimick breed formation, a bottleneck was introduced by gradually decreasing the population size to 75 males and 75 females during the next five generations. This population size was maintained for 95 generations, and, then, population size was increased to 1050 (50 males and 1000 females) in the last historical generation. From the last historical generation, all males and females were randomly mated for 15 generations to create the current population. Litter size in the current population was 10, and at each generation all sires and dams were replaced to create non-overlapping generations. For all scenarios, the reference population consisted of 500 randomly sampled individuals from generation 14, and the validation population consisted of 2000 randomly sampled individuals from generation 15.

### Genome

The genome consisted of 10 chromosomes, of 100 cM each. For each chromosome, 40 000 biallelic loci were simulated. Mutation rate in the historical generations was $2.5\cdot 10^{-6}$, and there was no mutation in the last 15 generations. From all loci segregating in generation 14, *m* loci were selected to become QTL, which varied across scenarios, and 5000 loci were selected to become markers. Although this density is lower than a typical commercial livestock SNP chip (60K), we chose this lower density to decrease computational demand. The markers were selected based on their allele frequency; the allele frequency distribution of markers was approximately uniform. The QTL were randomly selected and the allele frequency distribution of QTL was approximately U-shaped.

#### QTL effects

Additive effects (*a*) of QTL were sampled from a normal distribution with mean 0 and variance 1. Dominance factors ($\delta$) were also sampled from a normal distribution, with varying mean and variance across scenarios. Dominance effects (*d*) were computed as $\delta \left| a\right|$ [32, 33]. Similar to dominance effects, we assumed that the magnitude of epistatic effects were proportional to the additive effects of the interacting QTL. For all $m(m-1)/2$ pairwise combinations of QTL, epistatic factors ($\gamma$) were sampled from a normal distribution with mean 0 and variance 1. The epistatic effects ($\epsilon$) between QTL *k* and *l* were computed as $\gamma \sqrt{\left| a_k a_l\right| }$.

### Breeding values, dominance deviations, epistatic deviations, and phenotypes

**Fig. 2:**
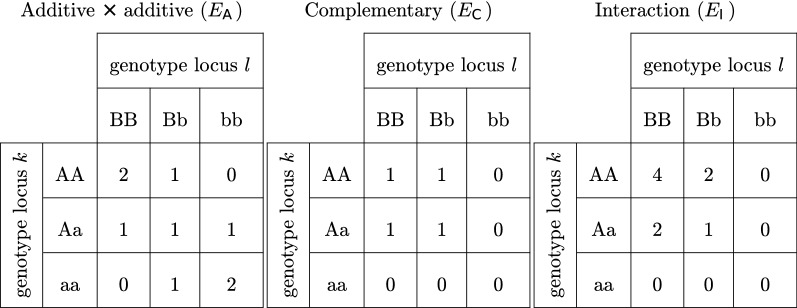
Epistatic models Epistatic coefficients used for simulating the total genetic values in three epistatic scenarios: additive by additive, complementary, and interaction

Breeding values ($\mathbf {A}$) and dominance deviations ($\mathbf {D}$) were simulated with genotype coefficient matrices that followed the natural and orthogonal interactions (NOIA) parameterization, as in [20]. With NOIA, the coefficient matrices are constructed such that the genetic effects ($\mathbf {A}$ and $\mathbf {D}$) are statistically orthogonal, even in the absence of HWE. However, the epistatic values were simulated with epistatic coefficient matrices that followed one of three biological models for epistasis (Fig 2). The resulting epistatic values are not orthogonal to $\mathbf {A}$ and $\mathbf {D}$, which means that $\mathbf {A}$ and $\mathbf {D}$ change when epistasis is simulated. Thus, we begin by explaining the simulation of epistatic deviations and subsequently discuss how $\mathbf {A}$ and $\mathbf {D}$ were computed.

The first step was to compute epistatic values for all nine possible combinations of genotypes at loci *k* and *l* as $\mathbf {c}_{kl}=\mathbf {t}\epsilon _{kl}$, where $\epsilon _{kl}$ is the epistatic effect between loci *k* and *l*, and $\mathbf {t}$ is a vector containing 9 ($3\times 3$) epistatic coefficients, following one of three epistasis models (Fig. 2). The coefficients in $\mathbf {t}$ were ordered from top-to-bottom and left-to-right (AABB, AaBB, aaBB, ..., aabb). Then, using the NOIA parameterization and the two-locus genotype frequencies, epistatic values were partitioned into nine statistically orthogonal effects following the procedure described in [20]:

$$\begin{aligned}&\mathbf {b}_{kl} = \left[ \mu , \alpha _{kl}^k, d_{kl}^k, \alpha _{kl}^l, \left( \alpha \alpha \right) _{kl}, \left( d\alpha \right) _{kl}, d_{kl}^l \left( \alpha d\right) _{kl}, \left( dd\right) _{kl}\right] ^{\intercal } \end{aligned}$$

This procedure was repeated for all $m(m-1)/2$ pairwise interactions between QTL.

The epistatic deviation of individual *i* was computed as:

$$\begin{aligned} E_i= & {} \sum _{{\mathop {k\ne l}\limits ^{k,l=1}}}^m h^k_{a,i}h^l_{a,i}(\alpha \alpha )_{kl} \\&+ h^k_{a,i}h^l_{d,i}(\alpha d)_{kl} + h^k_{d,i}h^l_{a,i}(d\alpha )_{kl}\\&+ h^k_{d,i}h^l_{d,i}(dd)_{kl}, \end{aligned}$$

where $h^k_{a,i}$ ($h^l_{a,i}$) is the additive genotype coefficient of individual *i* at locus *k* (*l*), and $h^k_{d,i}$ ($h^l_{d,i}$) is the dominance genotype coefficient of individual *i* at locus *k* (*l*). Elements of the additive genotype coefficients, $h^k_{a,i}$, were encoded as in (7), where $p_{AA}$, $p_{Aa}$, and $p_{aa}$ are the genotype frequencies of marker *k* in the base generation (generation 14). Elements of the dominance genotype coefficients were encoded as in (8). The breeding value of individual *i* was computed as:

$$\begin{aligned} BV_i = \sum _{k=1}^m h^k_{a,i} \alpha ^k, \end{aligned}$$

where $\alpha ^k$ is the average effect of locus *k*, which was computed as:

$$\begin{aligned} \alpha ^k = a^k + \left( 1-2p^k\right) d^k + \sum _{{\mathop {l\ne k}\limits ^{l=1}}}^m \alpha ^k_{kl}, \end{aligned}$$

where $p^k$ is the allele frequency of locus *k* in generation 14. The dominance deviation of individual *i* was computed as:

$$\begin{aligned} D_i = \sum _{k=1}^m h^k_{d,i} {d^k}', \end{aligned}$$

where ${d^k}'$ was computed as:

$$\begin{aligned} {d^k}' = d^k + \sum _{{\mathop {l\ne k}\limits ^{l=1}}}^m \alpha ^k_{kl}. \end{aligned}$$

Total genetic values were computed as $\mathbf {TGV} = \mathbf {BV} + \mathbf {D} + \mathbf {E}$. Phenotypes were computed as $\mathbf {y}= \mathbf {TGV} + \mathbf {e}$, where $\mathbf {e}$ is a vector of random residuals, sampled from a normal distribution with mean zero and variance $\sigma _e^2=\sigma _{TGV}^2$, such that the broad sense heritability $H^2$ is equal to $50\%$.

### Scenarios

As a base scenario, a purely additive trait with 300 QTL was simulated (Base). We varied the number of QTL to be 1000 ($S_{1000}$, 100 ($S_{100}$), or 10 $(S_{10}$). Hereafter, we will call this characteristic of the trait “Sparsity”. Dominance was varied by sampling dominance factors $\delta$ from $\mathcal {N}\left( 0.6,0.3^2\right)$ with the $D_{\text {medium}}$ scenario, or from $\mathcal {N}(1.2,0.3^2)$ with the $D_{\text {extreme}}$ scenario, which is extreme overdominance.

Following [1, 34], epistasis was varied by applying the additive $\times$ additive model ($E_{A}$), complementary model ($E_{C}$), or interaction model ($E_{I}$). The relative variance components in the simulated scenarios are listed in Table 1. The location and additive effects of QTL in each scenario were not resampled for the dominance and epistasis scenarios, so they were the same as in the base scenario.

**Table 1:** Summary of the scenarios used in the simulations

	#QTL	Explanation	Var(*A*)	Var(*D*)	Var(*E*)
Base	300	Default scenario	1.0	0.0	0.0
$S_{10}$	10	Very sparse	1.0	0.0	0.0
$S_{100}$	100	Sparse	1.0	0.0	0.0
$S_{1000}$	1000	Dense	1.0	0.0	0.0
$D_{\text {medium}}$	300	Medium dominance	0.854	0.161	0.0
$D_{\text {extreme}}$	300	Extreme dominance	0.636	0.386	0.0
$E_{A}$	300	Additive $\times$ additive epistasis	0.657	0.0	0.366
$E_{C}$	300	Complementary epistasis	0.658	0.225	0.116
$E_{I}$	300	Interaction epistasis	0.896	0.0	0.127

The rightmost columns contain the average proportions of additive, dominance, and epistatic variance in the replicate genotypes. In all scenarios, the broad sense heritability is $H^2=50\%$

### Comparison of methods

To evaluate the performance of the different methods, each one was trained on the 500 animals in the training population, and the accuracy was obtained by taking the Pearson correlation coefficient between predictions and the total genotypic values of the 2000 animals in the validation population.

Direct comparison of the average accuracies per scenario (Table 2) required many replicates, because the accuracies fluctuated considerably between replicates. Therefore, instead of comparing the average accuracies of the methods, we used the mean and standard error of the difference in accuracy between methods, $\rho _{\text {NetSparse}}-\rho _{\text {Method}}$, which fluctuated much less (Table 3). In addition, we calculated the *p*-values corresponding to the one-sided paired *t*-test for the null hypotheses ${\mathcal {H}}_0:\mathbb {E}\left( \rho _{\text {NetSparse}}-\rho _{\text {Method}}\right) =0$ for each reference method. Significance of *p*-values with respect to the treshold of 0.05 were corrected for multiple testing via the Benjamini-Hochberg procedure (Table 4).

**Table 2:** Mean accuracy and standard error of the mean of each method, calculated over ten replicates each, times 100

Scenario	GBLUP	BayesB	NetSparse	GBLUP-AD	BayesB-AD	GBLUP-ADAA
Base	$63.6\pm 1.2$	$63.8\pm 1.2$	$64.8\pm 1.4$	$62.5\pm 1.3$	$61.5\pm 1.5$	$62.2\pm 1.4$
$S_{10}$	$63.6\pm 1.5$	$84.6\pm 1.5$	$91.3\pm 1.3$	$63.1\pm 1.3$	$82.0\pm 1.4$	$62.7\pm 1.4$
$S_{100}$	$61.6\pm 0.7$	$64.4\pm 1.0$	$66.8\pm 1.3$	$61.0\pm 0.6$	$61.5\pm 1.1$	$60.7\pm 0.7$
$S_{1000}$	$66.0\pm 1.8$	$64.9\pm 1.7$	$66.0\pm 1.8$	$65.7\pm 1.8$	$62.9\pm 2.1$	$65.5\pm 1.8$
$D_{\text {medium}}$	$55.4\pm 2.0$	$55.6\pm 1.8$	$56.2\pm 2.1$	$56.1\pm 2.0$	$55.4\pm 2.2$	$55.8\pm 2.0$
$D_{\text {extreme}}$	$42.8\pm 1.7$	$42.8\pm 1.5$	$43.4\pm 1.8$	$49.7\pm 1.7$	$49.5\pm 1.7$	$49.2\pm 1.7$
$E_A$	$43.9\pm 2.0$	$44.0\pm 1.8$	$44.6\pm 2.1$	$43.2\pm 2.2$	$41.4\pm 2.0$	$43.3\pm 2.4$
$E_C$	$44.9\pm 2.1$	$44.5\pm 2.4$	$45.4\pm 2.4$	$44.5\pm 2.1$	$43.9\pm 2.4$	$44.4\pm 2.2$
$E_I$	$56.5\pm 1.5$	$56.4\pm 1.7$	$58.0\pm 1.6$	$55.2\pm 1.5$	$54.0\pm 1.8$	$55.2\pm 1.5$

Each row corresponds to a scenario, as summarized in Table 1. The different columns correspond to different methods, GBLUP and Bayes-B are additive methods, GBLUP-AD and BayesB-AD are methods with additive and dominance features and GBLUP-ADAA has additive, dominance and additive × additive features

**Table 3:** Mean accuracy increase of NetSparse relative to each other method and its standard error on the mean calculated over ten replicates each, times 100

Scenario	GBLUP	BayesB	GBLUP-AD	BayesB-AD	GBLUP-ADAA
Base	$1.2\pm 0.6$	$1.0\pm 0.5$	${\textit{2.2}\pm \textit{0.7}}$	${\textit{3.3}\pm \textit{0.6}}$	${\textit{2.5}\pm \textit{0.6}}$
$S_{10}$	${\textit{27.7}\pm \textit{1.6}}$	${\textit{6.7}\pm \textit{0.8}}$	${\textit{28.1}\pm \textit{1.5}}$	${\textit{9.3}\pm \textit{1.1}}$	${\textit{28.5}\pm \textit{1.4}}$
$S_{100}$	${\textit{5.2}\pm \textit{1.1}}$	${\textit{2.4}\pm \textit{0.7}}$	${\textit{5.8}\pm \textit{1.1}}$	${\textit{5.4}\pm \textit{0.7}}$	${\textit{6.1}\pm \textit{1.1}}$
$S_{1000}$	$-0.0\pm 0.2$	${\textit{1.1}\pm \textit{0.3}}$	$0.3\pm 0.2$	${\textit{3.0}\pm \textit{0.7}}$	$0.4\pm 0.3$
$D_{\text {medium}}$	${\textit{0.8}\pm \textit{0.3}}$	$0.6\pm 0.5$	$0.0\pm 0.5$	$0.8\pm 0.6$	$0.4\pm 0.5$
$D_{\text {extreme}}$	${\textit{0.6}\pm \textit{0.8}}$	$0.6\pm 0.6$	$-6.3\pm 0.6$	$-6.1\pm 0.7$	$-5.8\pm 0.7$
$E_A$	${\textit{0.7}\pm \textit{0.2}}$	$0.6\pm 0.7$	${\textit{1.4}\pm \textit{0.4}}$	${\textit{3.2}\pm \textit{0.6}}$	${\textit{1.3}\pm \textit{0.5}}$
$E_C$	$0.6\pm 0.4$	${\textit{0.9}\pm \textit{0.3}}$	$0.9\pm 1.0$	$1.5\pm 1.1$	$1.0\pm 1.0$
$E_I$	${\textit{1.5}\pm \textit{0.5}}$	${\textit{1.5}\pm \textit{0.4}}$	${\textit{2.8}\pm \textit{0.6}}$	${\textit{3.9}\pm \textit{0.6}}$	${\textit{2.7}\pm \textit{0.6}}$

Significant entries, determined with the Benjamini-Hochberg procedure for $\alpha =0.05$ for the one-sided paired *t*-test corresponding to the hypotheses $\mathbb {E}\left( \rho _{\text {NetSparse}}-\rho _{\text {Method}}\right) =0$, are marked in italic

**Table 4:** *p*-values of the one-sided paired *t*-test for the hypotheses $\mathbb {E}\left( \rho _{\text {NetSparse}}-\rho _{\text {Method}}\right) =0$

Scenario	GBLUP	BayesB	GBLUP-AD	BayesB-AD	GBLUP-ADAA
Base	$3.21\times 10^{-2}$	$3.98\times 10^{-2}$	$4.52\times 10^{-3}$	$1.92\times 10^{-4}$	$1.67\times 10^{-3}$
$S_{10}$	$2.05\times 10^{-8}$	$6.96\times 10^{-6}$	$7.45\times 10^{-9}$	$9.36\times 10^{-6}$	$4.48\times 10^{-9}$
$S_{100}$	$4.63\times 10^{-4}$	$3.64\times 10^{-3}$	$2.18\times 10^{-4}$	$2.79\times 10^{-5}$	$1.52\times 10^{-4}$
$S_{1000}$	$5.00\times 10^{-1}$	$3.48\times 10^{-3}$	$1.33\times 10^{-1}$	$1.29\times 10^{-3}$	$9.67\times 10^{-2}$
$D_{\text {medium}}$	$5.75\times 10^{-3}$	$1.47\times 10^{-1}$	$5.00\times 10^{-1}$	$1.19\times 10^{-1}$	$2.10\times 10^{-1}$
$D_{\text {extreme}}$	$2.09\times 10^{-2}$	$1.72\times 10^{-1}$	$-1.85\times 10^{-6}$	$-7.57\times 10^{-6}$	$-5.45\times 10^{-6}$
$E_A$	$5.75\times 10^{-3}$	$1.95\times 10^{-1}$	$1.50\times 10^{-3}$	$2.39\times 10^{-4}$	$1.14\times 10^{-2}$
$E_C$	$8.92\times 10^{-2}$	$9.61\times 10^{-3}$	$1.98\times 10^{-1}$	$1.04\times 10^{-1}$	$1.60\times 10^{-1}$
$E_I$	$7.92\times 10^{-3}$	$1.67\times 10^{-3}$	$4.13\times 10^{-4}$	$8.27\times 10^{-5}$	$5.27\times 10^{-4}$

Each row corresponds to a scenario, as summarized in Table 1. The cells containing negative values had *p* close to 1 and therefore we chose to put $-(1-p)$ in those cell instead. The minus serves to clearly identify those cases while the $(1-p)$ represents the *p*-value for superiority of GBLUP-AD and BayesB-AD over NetSparse

## Results

**Fig. 3:**
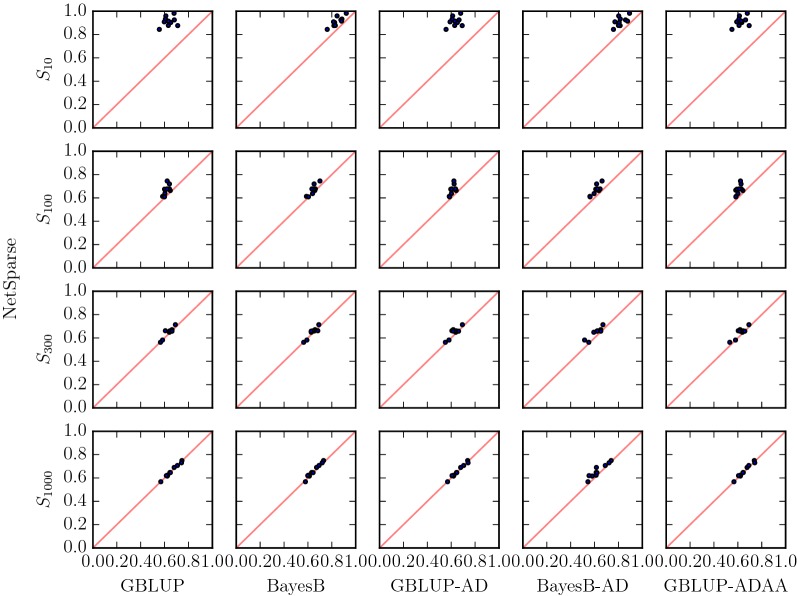
Sparsity The accuracy of NetSparse versus other methods in scenarios with 10, 100, 300 and 1000 QTL. Each row corresponds to a different amount of sparsity, the different columns correspond to different methods, GBLUP and Bayes-B are additive methods, GBLUP-AD and BayesB-AD are methods with additive and dominance features and GBLUP-ADAA has additive, dominance and additive$\times$additive features. The line $x=y$ is added in red for reference. A marker that is above the line means a replicate with higher accuracy for NetSparse than the method it is compared to, and a marker that is below the line means a replicate with lower accuracy for NetSparse than the other method

First, we considered the effect of sparsity on the prediction of genotypic values in the additive scenarios for all methods (Fig. 3). In the sparse scenario with 10 QTL ($S_{10}$), the accuracy with Netsparse was about 0.28 higher than with GBLUP(-AD,-ADAA), and about 0.08 higher than with BayesB(-AD). In the scenario with 100 QTL ($S_{100}$), NetSparse had an increase in accuracy of  0.06 over the GBLUP(-AD,-ADAA) methods, of  0.02 over BayesB and 0.05 over BayesB-AD. In the “Base” scenario with 300 QTL, NetSparse was better than the methods that fit dominance, but not significantly better than the additive methods In the 1000 QTL scenario NetSparse was significantly better than BayesB and BayesB-AD, but not significantly better than the methods based on GBLUP.

**Fig. 4:**
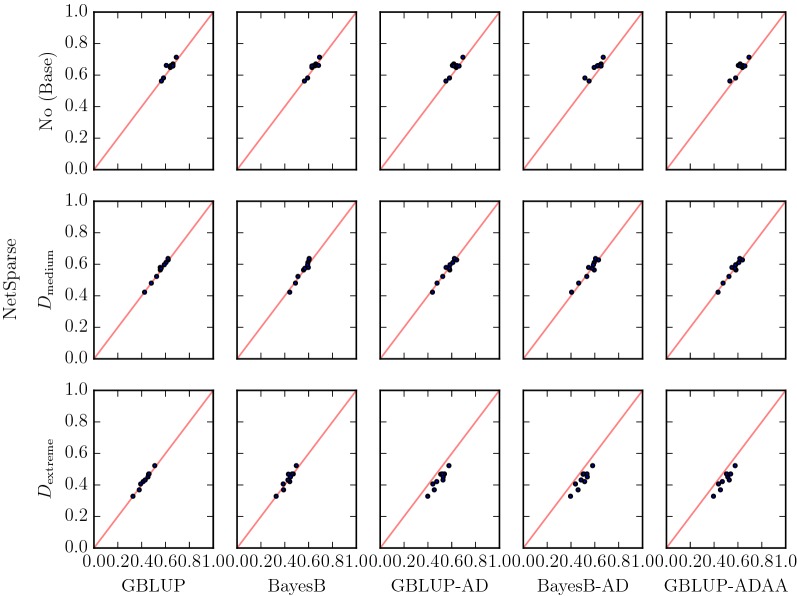
Dominance Accuracy of NetSparse versus other methods for the base scenario, and the two (Medium and Extreme) dominance scenarios. The line $x=y$ is added in red for reference. A marker above the line means a replicate with higher accuracy for NetSparse than the method it is compared to, a marker below the line means a lower accuracy of NetSparse than the other method

Now, we consider the simplest possible phenotypic model after the additive one, the dominance model. In the medium dominance scenario ($D_{\text {medium}}$), all methods performed roughly the same (Fig. 4). Hence, methods that tried to fit dominance did not result in higher accuracies than methods that did not. In the extreme dominance scenario ($D_{\text {extreme}}$), GBLUP-AD, BayesB-AD and GBLUP-ADAA methods had better performance than the other methods, which matched our prior expectation.

**Fig. 5:**
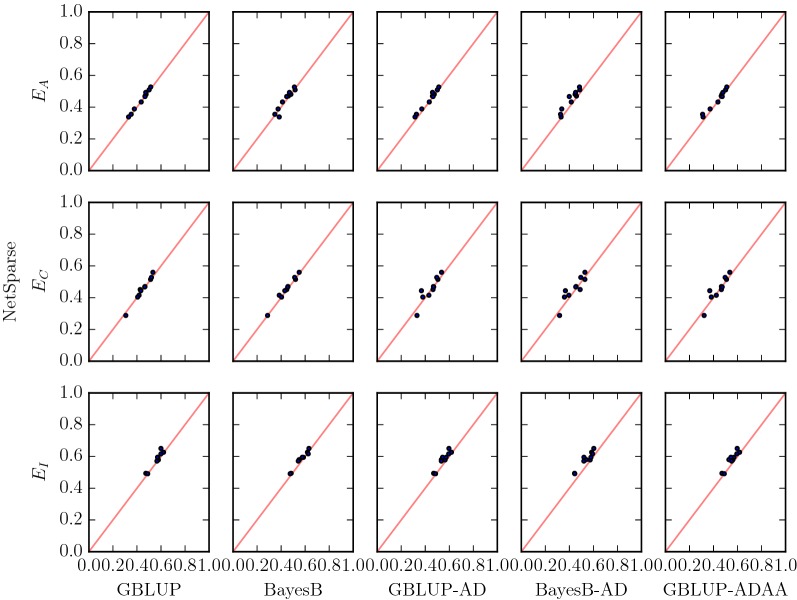
Epistasis Accuracy of NetSparse versus other methods for the three epistatic scenarios: Additive $\times$ Additive, Complementary and Interaction. The line $x=y$ is added in red for reference. A marker above the line means a replicate with higher accuracy for NetSparse than the method it is compared to, a marker below the line means a lower accuracy of NetSparse than the other method

The epistatic scenarios (Fig. 5) contain components which can be fitted only by NetSparse and GBLUP-ADAA, thus we expected that in the additive $\times$ additive scenario, GBLUP-ADAA would have the best fit and that NetSparse would have the best fit among the other two scenarios. In the additive $\times$ additive scenario ($E_A$), NetSparse had a significantly higher accuracy than the other methods except BayesB. Surprisingly, GBLUP-ADAA did not fit this scenario better than the other methods. In the complementary ($E_C$) scenario, NetSparse had 0.6 to 1.5 percentage points higher accuracy on average than the other methods, but these results were not consistent across replicates. The accuracy of NetSparse in the interaction scenario ($E_I$) was on average three or more standard errors above the other methods.

## Discussion

In this study, we compared methods that differed in flexibility. For example, the GBLUP-AD method is more flexible than the GBLUP method, because it allows for dominance effects to be fitted. In theory, the more flexible method should be able to give the same predictions as simpler methods by setting the additional hyperparameters to zero. In reality, however, these additional hyperparameters also have to be estimated from the data because the genetic architecture of the trait is unknown. In this study, we used the default prior distributions from the BGLR package and estimated hyperparameters from the training dataset. We chose not to fine-tune the prior distributions on test performance, because in reality this is not possible. As a result, when the actual genetic architecture of a trait is simple (e.g. additive and not sparse), a more flexible method will perform worse than a simpler method. Our results indeed showed that sometimes more flexible methods performed worse than simpler methods. For example, if we consider the scenario with complementary epistatic effects and consider the GBLUP and BayesB methods, BayesB with hyperparameter $\pi$ set to zero is equivalent to GBLUP, but when fitting the value of $\pi$ in BayesB to the data, a non-zero value of $\pi$ is estimated, which in this scenario gives worse test performance than $\pi =0$. In [5], it also was seen that, in certain cases, GBLUP can have higher accuracy than BayesC, which is a sparse method similar to BayesB.

**Fig. 6:**
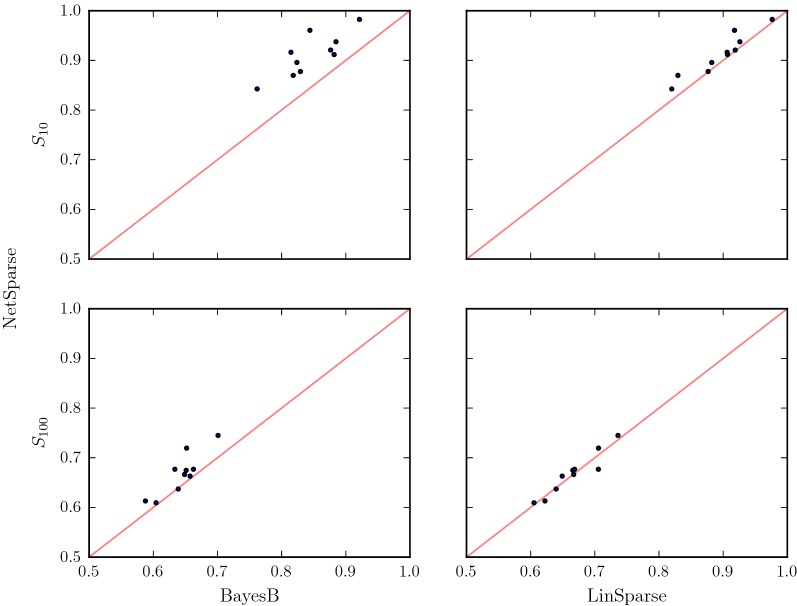
Sparsity The accuracy of NetSparse versus BayesB and LinSparse on sparse scenarios with 10 or 100 QTL. The line $x=y$ is added in red for reference. A marker above the line means a replicate with higher accuracy for NetSparse than the method it is compared to, a marker below the line means a lower accuracy of NetSparse than the other model

The particular observation that NetSparse has higher accuracy than BayesB for the $S_{10}$ and $S_{100}$ scenarios was unexpected because BayesB is a sparse additive method, while NetSparse is a sparse non-additive method. Since the underlying data generating process is sparse additive, the expectation is that BayesB matches the simulated data better than NetSparse. The difference in method between NetSparse and BayesB is that NetSparse includes non-additivity and that NetSparse and BayesB use different priors for the variances. Therefore, we also made a comparison with LinSparse (Fig. 6), which is NetSparse without non-additive effects. The accuracy obtained with LinSparse for these scenarios was higher than for BayesB, which strongly suggests that the difference in accuracy between them originated from the different prior distributions for the variances.

In BayesB, the prior distributions for the variances are scaled inverse chi-squared distributions, which are conjugate priors for the likelihood function, which makes Gibbs sampling possible. The NUTS sampler in PyMC3 does not require conjugate priors and, following the suggestions of [35], we chose half-normal distributions for the standard deviations. The main difference between the scaled inverse chi-squared and half-normal distributions is that the half-normal distribution decays faster than exponentially for large values, which gives much lighter tails than the scaled inverse chi-squared distribution, which decays only polynomially.

The epistatic method GBLUP-ADAA did not seem to give better fits than methods that did not fit epistasis. We think this is due to a lack of data for estimating epistatic effects accurately. Inaccurate estimates of these effects will not improve predictive ability.

Given that neural networks are able to fit more than additive relations, we expected that NetSparse would also be able to fit dominance and epistatic effects. This expectation was confirmed in scenarios with strong dominance effects and scenarios with epistatic effects because accuracy of NetSparse was higher than the accuracy of additive models. However, NetSparse may be at a disadvantage with traits that have negligible non-additive effects. Nevertheless this indicates a potential for Sparse Bayesian Neural Networks for improving phenotype prediction.

The main limitation of NetSparse is running time. On our hardware, training NetSparse took around 4 h per scenario with 500 animals and 5000 SNPs. The running time of NetSparse scales approximately linearly with both the number of animals and the number of SNPs, and can therefore become prohibitive when applied to larger datasets. The other methods have running times that were less than 2 min on these datasets, making them much more feasible for use on larger datasets. Considering the promising results of NetSparse, further studies could try to increase the computational performance so that larger datasets can be analyzed. As sampling of independent MCMC chains can be done in parallel on different machines, additional computational resources can speed up the wall time of sampling by a factor equal to the number of chains used. The MCMC sampling could also be replaced by variational inference, where the real posterior is approximated by a simpler variational posterior from which samples can be drawn directly, which would help NetSparse scale to larger datasets. The discrete variable $\mathbf {s}$ could be handled, for instance, using the Concrete Distribution. [36]

## Conclusions

This study shows that in nearly all scenarios the accuracy of NetSparse is not significantly lower than that of all other methods investigated. In particular, the NetSparse method performed as well or better than GBLUP and BayesB for all scenarios evaluated. On data generated from a sparse QTL simulation model, accuracies obtained with NetSparse were significantly higher than accuracies obtained with all the other methods investigated. In the medium dominance scenarios, accuracy obtained with NetSparse was 0.0 to 0.8 percentage points higher than that with the other methods investigated. In the extreme dominance scenario, accuracy obtained with Netsparse was 0.6 percentage points higher than that with other methods that did not explicitly model dominance. For methods that did explicitly model dominance, the accuracy was 5.8 to 6.3 percentage points lower for NetSparse. In the epistatic scenarios, accuracy obtained with NetSparse was 0.6 to 3.9 percentage points higher than that with the other methods. However, running time can be limiting, as NetSparse inference took about 200 times as long as the other methods.

## Data Availability

Not applicable.

## Appendix A: Prior distributions

The prior distributions for NetSparse are:

$$\begin{aligned} e_i \mid \beta&\sim \mathcal {N}\left( 0, \beta ^{-1}\right) ,\\ W_{ij}\mid \sigma _h&\sim \mathcal {N}\left( 0,\sigma _h^2/P\right) ,\\ \mathbf {w}\mid \sigma _o&\sim \mathcal {N}\left( 0,\sigma _o^2/H\mathbf {1}\right) ,\\ \mathbf {b}^h&\sim \mathcal {N}\left( \varvec{\upmu }^h, \sigma _{b_h}^2\mathbf {1}\right) ,\\&\big (\varvec{\upmu }^h= [-2\sigma _h,\ldots , 2\sigma _h],\\&\quad \sigma _{b_h} = 4\sigma _h/\sqrt{H} \big )\\ b^o&\sim \text {Unif}\left( -\infty ,\infty \right) ,\\ \beta , \sigma _h, \sigma _o&\sim \left| \mathcal {N}\left( 0,2^2\right) \right| ,\\ s^p\mid \pi&\sim \text {Ber}(1-\pi ) \text {, and}\\ \pi&\sim \text {Unif}\left( 0,1\right) . \end{aligned}$$

The prior distributions for LinSparse ($f(\mathbf {x};\mathbf {u})=\mathbf {w}^{\intercal }\left( \mathbf {x}\odot \mathbf {s}\right) + b$) are:

$$\begin{aligned} e_i \mid \beta&\sim \mathcal {N}\left( 0, \beta ^{-1}\right) ,\\ \mathbf {w}\mid \sigma&\sim \mathcal {N}\left( 0,\sigma ^2/P\mathbf {1}\right) ,\\ b&\sim \text {Unif}\left( -\infty ,\infty \right) ,\\ \beta , \sigma&\sim \left| \mathcal {N}(0,2^2)\right| ,\\ s^p\mid \pi&\sim \text {Ber}(1-\pi ) \text { and}\\ \pi&\sim \text {Unif}\left( 0,1\right) . \end{aligned}$$

The prior distributions of $b^h_i$ each have a different mean because equal priors would make the model invariant under a relabeling of the hidden units and result in a degenerate geometry of the sampling space where each $\mathbf {u}$ is equivalent to at least $H!-1$ other configurations. For 20 hidden units, there are over $10^{18}$ configurations, which makes it completely infeasible to explore the entire parameter space.

We could analytically marginalize out $\sigma _o$ and $\sigma _h$, resulting in a probability density over $\mathbf {w}$ in terms of the modified Bessel function of the second kind:

$$\begin{aligned} p\left( \mathbf {w}\right) \propto \left\| \mathbf {w}\right\| ^{(1-H)/2} K_{(H-1)/2}\left( \frac{\sqrt{H}}{2}\left\| \mathbf {w}\right\| \right) \end{aligned}$$

and similarly for $\mathbf {W}$. For simplicity, we kept the model parameterization in terms of $\sigma _h$ and $\sigma _o$.

## Footnotes

1 Strictly speaking, in GBLUP the allele effects of SNP-BLUP are integrated out, so the variance components in GBLUP should be called “parameters”. But for consistency with the other methods, we refer to the variance components as hyperparameters.

2 Because $\mathbf {s}$ is discrete it is summed instead of integrated over.

3 As activation functions were applied to layers, one in hidden and one in output layer, this architecture is also called a two-layer neural network.

4 A complete list of prior distributions is given in appendix A.
